# Right Ventricular Evaluation with Speckle Tracking Echocardiography in COPD after a Pulmonary Rehabilitation Program

**DOI:** 10.5935/abc.20180183

**Published:** 2018-09

**Authors:** Djair Brindeiro Filho

**Affiliations:** Serviço de Ecocardiografia do Recife (SECOR) - Real Hospital Português de Beneficência em Pernambuco, Recife, PE - Brazil

**Keywords:** Chronic obstructive pulmonary disease (COPD), Two-dimensional Echo, Strain, Speckle Tracking, Pulmonary Rehabilitation

Chronic obstructive pulmonary disease (COPD) is a serious public health problem, and is often related to smoking.^1^ In advanced stages of COPD, the presence of PAH is a common development. PAH progression rate in COPD is usually slow (an increase of < 1 mmHg per year). However, the presence of even moderate PAH is a strong predictor of mortality.^2^ During stable periods of the disease, the increase in mean pulmonary artery pressure is usually mild to moderate. However, severe PAH may occasionally occur in COPD patients COPD.^3^

Conventional two-dimensional (2D) echo parameters allow a reasonable assessment of RV Function. In the 1990s, the use of tissue Doppler (TD) to measure the intramyocardial velocity gradient allowed measuring the rate of myocardial strain and its percentage (strain rate and strain). About ten years ago, the speckle tracking technique, based on the tracking of the speckles which two-dimensional echo images, allowed assessing myocardial strain without limitation by the DT insonation angle.^4^ 2D-STE strain can not only quantify the overall RV function, but it can also identify discrete, localized contractile losses, providing information regarding the pathophysiological mechanisms that lead to right ventricular failure.^5^ In a heterogeneous group of patients, RV lateral wall longitudinal strain showed a strong correlation with RV ejection fraction calculated by cardiac magnetic resonance.^6^ Peak longitudinal strain is a significant prognostic determinant in PAH patients, with greater value compared to other known clinical and echocardiographic predictors of mortality.^7^

Several studies have used 2D-STE in chronic PAH patients. Several authors advocate this method for serial evaluation of PAH patients since RV free-wall strain has proved an independent predictor of clinical deterioration and mortality after medical therapy is initiated.^8^ Although 2D-STE is widely used in various clinical conditions, the guidelines on echocardiographic evaluation of RV function strongly recommends including other measures into echocardiographic examination and report.^9^ In addition to the fact that there are no reference values, RV ST2D can be influenced by image quality, reverberation and other artifacts.^10^

The literature clearly shows the benefit of pulmonary rehabilitation (PR) programs. A prospective randomized study showed the effectiveness of respiratory training as an additional treatment of severe chronic PAH.^11^ In this issue, Kanar et al. evaluated the RV function using 2D-STE with 46 COPD and 32 control patients.^12^ The authors compared the 2D-STE values for the two groups and for patients before and after a pulmonary rehabilitation program. The conventional parameters for 2D-echo and 2D-STE showed a similar correlation between COPD and control patients, but RV longitudinal strain showed greater sensitivity in examining the relationship between RV function and exercise tolerance. The main limitations are pointed out in the article. There is no information on whether 2D-STE measurements were made in apnea or at the time of pre and post PR breathing. Since RV is sensitive to preload variations, the values could be influenced by respiratory variation. In any case, the usefulness of ST2D to evaluate RV in COPD was well demonstrated. Although there is controversy on the effectiveness of pulmonary rehabilitation programs in PAH,^13^ the authors demonstrated in an original way, i.e., through ST2D, that RV improves after PR, thus creating new perspectives for the use of PR in COPD patients.
